# Effects of Plant Metabolites on the Growth of COVID-19 (Coronavirus Disease-19) Including Omicron Strain

**DOI:** 10.7759/cureus.26549

**Published:** 2022-07-04

**Authors:** Hiroj Bagde, Ashwini Dhopte

**Affiliations:** 1 Periodontology, Rama Dental College and Research Centre, Kanpur, IND; 2 Oral Medicine and Radiology, Rama Dental College and Research Centre, Kanpur, IND

**Keywords:** covid-19, antiviral activity, metabolites, medicinal plants, medication, therapeutic medicine

## Abstract

According to recent reports out of India, a new strain of Severe Acute Respiratory Syndrome Coronavirus-2 (SARS-CoV-2) B1.1.529 Omicron virus has emerged. In comparison to the Wuhan (WHU) strain and the delta variant, this variant showed a far stronger effect on the angiotensin converting enzyme2 (ACE2) receptor. There are several medicinal compounds in plant metabolites, and their diverse chemical structures make them ideal for the treatment of serious illnesses. It's possible that some of these could be useful alternative pharmaceuticals, as well as a starting point for the repurposing of existing medications and new chemical discoveries. SARS-CoV-2 infection triggered a worldwide epidemic of the severe acute respiratory syndrome (SARS). There have been trials for different therapies for SARS-CoV-2 and so also there are recent announcements of extensive research into the development of viable medicines for this global health calamity. After a thorough examination of plant-derived treatments for COVID-19, investigators in the current study decided to focus on plant-derived secondary metabolites (PSMs). According to some researchers, new MDR (Multi-Drug Resistant) antibiotics may one day be developed due to the adaptability of secondary metabolites. Identifying plant metabolites that can treat a wide range of viral infections was one of the study's aims. Many natural medications that could be recommended for the treatment of COVID-19 were discovered as a result of this research, including remedies from plant families, viral candidates that are susceptible, antiviral assays, and mechanisms of therapeutic action. The findings of this study will inspire further research and speed up the development of new antiviral plant-based medications.

## Introduction and background

Conferring to the WHO, the SARS-CoV-2 B1.1.529 Omicron strain was discovered in Botswana and first reported in India on December 24 [1-8]. When compared to the WHU strain and the delta variation, this variant had stronger angiotensin converting enzyme2 (ACE2) receptor connections. Strain-specific immunological protection is a pipe dream in contexts where present vaccination coverage, cross-protection, and herd immunity are unsolved, making strain-specific immunological protection impossible [B1.1.529]. Between days 189 and 217, SARS-CoV-2 titers increased by 54.4-fold (Ancestral), 21.9-fold (Alpha), 24.5-fold (Beta), 24.4-fold (Delta), and 20.1-fold (Delta), respectively (Omicron) [3-9].

A comparison of the anti-rS IgG activity of the same SARS-CoV-2 strains was made at the same time points across the same period of time [10-11]. The recently discovered Omicron strain (B1.1.529) poses such a big threat, everyone is wondering when this epidemic will come to an end [12-14]. Discovered on November 11, 2021, the substantially changed Omicron strain originated in Botswana and South Africa. The substantial number of mutations that have occurred in Omicron's spike protein compared to the original Wuhan strain have reduced its binding efficacy to the ACE2 receptor. The hot-spot interaction between B1.1.529 and hACE2 has been shifted from its prior location in this release. Humans, bats, and other mammals are infected with noroviruses, which spread through the respiratory tracts. the 2019 new coronavirus (2019-nCoV), a rapidly spreading respiratory infection that was first discovered in the Chinese city of Wuhan, is now known as the Coronavirus Disease 2019 (COVID-19) [15-17]. The World Health Organization (WHO) has declared it a global pandemic because it has spread to 216 countries and killed more than 0.5 million people.

So far, human coronaviruses have been discovered in seven different forms, including the human coronavirus (HCoV) OC43, the HCoV-229E, the human coronavirus (HC) VHKU1, the HCVHKU2, the HCVHKU3, the SARS-CoV-2, the Middle East Respiratory Syndrome coronavirus (MERS-CoV), and the newly discovered virus in 2019 [18-20]. The viruses MERS-CoV, SARS-CoV-2, and novel coronavirus (nCoV), to name just a few, have sparked the interest of virus scientists all around the world. As a result of a SARS outbreak in Guangdong in 2003, 8,000 individuals were infected and 800 people died throughout 26 different countries [20-24]. Only a decade later, another coronavirus wreaked havoc around the world, leading to the MERS pandemic, which sickened 2,494 people and claimed the lives of 858 [25-27]. When it came to the outbreak of the COVID-19 pandemic caused by the SARS CoV-2 virus, the results were devastating. China was the first country to be infected with SARS CoV-2 [28-30]. First, the virus spread to several countries outside of the United States. Then it spread to the United Kingdom. Patients with COVID-19 have a higher risk of death and morbidity because of pathobiology components such as the viral response and hyperbolic host reaction. With Brazil, Mexico, and Peru serving as new SARS CoV-2 hotspots in Latin America, the virus has spread to the region [31]. In severe COVID-19, cytokine levels (IL-6, IL-10, and TNF-), lymphopenia (in both CD4+ and CD8+ T cells), and decreased interferon (IFN) synthesis in CD4+ T cells are all related to higher cytokine levels, which are all life-threatening. A specific drug, vaccine, or other certified therapeutic treatment for SARS-CoV-2 has not been developed, resulting in substantial morbidity and mortality among persons who have been exposed to the virus [32-33]. As a result of their healing capabilities, medicinal plants have been essential to human health since the beginning of time [34-35]. Medical plants and herbs are used therapeutically by more than 80% of the global population, according to the World Health Organization [36-39]. Antiviral compounds produced from a wide variety of plants have been used in numerous studies, all of which have been found to be effective [40]. Chemicals developed from antiviral plant secondary metabolites (PSMs) and compounds generated from medicinal plants are being tested around the world to avert this global catastrophe. Papain-like and 3CL proteases, as well as ACE2 protein fusion, have been shown to interfere with CoV replication by plant metabolites according to National Cancer Institute research. Researchers are currently using PSMs to screen for new compounds produced by medicinal plants that could be useful to treat a variety of diseases, including COVID-19) [41-44].

As a result, the current review deals with all available plant-derived antiviral metabolites which are available as an alternative approach against COVID-19. In order to create targeted treatments for newly found diseases, researchers must first understand the structure, life cycle, pathogenicity, cell signaling, epidemiology, pharmaceutical targets, and drug discovery process of these viruses [45-46].

## Review

Epidemiology, molecular organization, and life cycle of the SARS CoV-2 virus

There has been an outbreak of SARS CoV-2 in China's Wuhan seafood market in December 2019, making it the most damaging virus epidemic since SARS CoV and MERS [47-49]. It has been found that the virus can spread by close contact with infected people or exposure to coughing, sneezing, and respiratory droplets. According to reports, it has spread to 216 countries and killed about 0.5 million people. Over 11 million people have been infected with SARS CoV-2 infected in the United States and its allies like Russia and the European nations of France, Italy, Germany, Spain, and the United Kingdom before it [50-55]. An RNA genome of between 26.4 and 31.7 kb, and a crown-like glycoprotein on its surface, characterizes SARS COV-2, which can be pleomorphic. SARS CoV is more closely linked to MERS than MERS is to SARS CoV. The RNA genome of CoV-2, on the other hand, is considered to be one of the largest among RNA viruses. One or more of the remaining four open reading frames (ORFs), including the envelope glycoprotein or spike protein (S), envelope (E), membrane (M), and nucleocapsid, encodes non-structural proteins, whereas the other four ORFs encode structural proteins. Host cell membrane permeability and virus-host cell interaction are mediated by the S and E proteins, respectively, respectively [56-64]. The nucleocapsid (N) protein is generally engaged in the processing of the helical rib nucleocapsid complex, which contains various auxiliary proteins, while the M protein is known as the coronavirus assembly's principal organizer [61-63, 65]. The SARS CoV-2 genome has been shown to include six different mutations, three of which have been found in the 1ab gene and two in the S gene. According to the results of a proteomic investigation, SARS CoV-2 and SARS Cove are quite similar, but two proteins are not. Upon entering the body, SARS CoV-2 attaches to the receptor-binding domain (RBD) via the virion's surface glycoprotein (Spike-protein), which then tries to activate the hACE2 receptor in order to complete its life cycle. For the SARS CoV-2 to enter the body, it requires the viral receptor, ACE2, as well as the cellular transmembrane serine protease 2 (TMPRSS2). However, the virus's envelope and capsid are destroyed when the SARS CoV-2 virion particle merges with the host cell membrane. For translation of ORF1a and ORF1ab polypeptides into the host cell's cytoplasm, the virus injects its genetic material (RNA), which functions as an mRNA. There are 16 non-structural proteins (NSP) that are involved in replication and transcription after 3CLpro removes them from the polypeptides. After being taken over by SARS CoV-2, infected cells begin to synthesize proteins. SARS CoV-2 is assembled into new virion particles by the immune system in this circumstance. Assembled viral nucleic acids and proteins are subsequently transported into the ERGIC lumen and expelled from the cells through exocytosis. The virions produced by infected cells spread to other types of human cells. All stages of SARS can be divided into three: asymptomatic, non-severe symptomatic, and severe infection [66,67].

Elevated levels of cytokines and chemokines have been observed in SARS CoV-2 patients; cytokines are especially high in patients admitted to intensive care units (ICU's). A patient's serious condition is brought on by these unnaturally high levels [68-70]. There are two forms of spike glycoprotein, the primary mediator of SARS CoV-2, and the enzyme 3CLpro of SARS-CoV-2 shares 99.02 percent sequence identity with 3CLpro of SARS-CoV, which is also quite similar to bat SARS CoV 3CLpro. A greater affinity for the host cell receptor is seen in SARS CoV-2 than in SARS CoV itself (194). The substrate-binding site of bat SARS CoV-2 has undergone significant strategic changes, and SARS CoV-2 has 12-point mutations compared to SARS CoV. Major hydrogen bonds are disrupted and the receptor binding site is altered by SARS-CoV-2 3CLpro mutations (RBS). SARS CoV-2 vaccine development is complicated by the fact that recurrent mutations can lead to new strains with different pathogenicity [71].

The main drug targets of SARS CoV-2

Multi-viral infections can be successfully treated by interfering the human host-virus interactions. Anti-COVID-19 drugs may find easy targets in the key structural proteins of SARS CoV-2. There is a total of 16 NSPs that can be examined as well. SARS CoV-2 vaccine/drug development is hampered by the occurrence of repetitive recombination events. Although SARS-CoV-2 and CoV identify the same receptor (ACE2) in humans, there is a considerable difference in antigenicity between SARS-CoV and SARS-CoV2, which has important implications for the development of therapeutic alternatives against this rapidly expanding virus. As with SARS-CoV, the spike protein of SARSCoV-2 has a higher affinity for the ACE2 receptor than does that of SARS-CoV, but the RBD (Receptor Binding Domain) of SARS-CoV-2 is less compatible with hACE2. TMPRSS2 and TMPRSS2 are two of the most important SARS CoV-2 enzymes, which are responsible for S-protein activation and are interesting targets for COVID therapy [72-74].

Effective therapeutics and SARS-CoV-2

Vaccines and pharmaceutical compounds against SARS-CoV-2 are being researched extensively, although no cures have yet been found. It's also possible to manage COVID-19 by using interferon treatments, monoclonal antibodies, oligonucleotides and small-molecule medications and vaccines. Coronavirus epidemics can be treated with existing drugs, but this is not the only option for eliminating the sickness. There's been a great deal of attention paid to developing therapeutic drugs for the COVID-19 outbreak. Researchers from a variety of fields are working together to come up with new therapeutic options. In contrast, testing ideas for systematic drug recombination may be difficult due to the high costs and long lead times associated with experiments resulting from drug recombination [75].

Control of SARS COV-2 with synthetic drugs

Licensed, repurposed drugs are currently being used in clinical trials, but focused antiviral treatments and vaccines are still urgently needed in the marketplace [75-76]. Bioengineered and vectored antibodies, cytokines, and nucleic acid-based medicines that target virus gene expression have all been discovered as promising treatments for coronavirus infections. Repurposing medications such favipiravir, remdesivir, lopinavir, ritonavir, nebulized-interferon, chloroquine, hydroxychloroquine, ribavirin, and interferon (IFN) have been demonstrated to be beneficial in the treatment of COVID-19, as well. Also being examined in clinical trials are additional drugs, including a peptide vaccine (mRNA-1273) and antibody treatment [77]. When it comes to the treatment of COVID-19, plasma therapy has recently shown promising outcomes [76]. A number of immunoinformatic approaches are being used by researchers throughout the world in an effort to identify some of the most promising treatment and vaccine candidates against this deadly virus. There is therefore an urgent requirement for COVID-19 therapies/drugs that are safe, effective, and reasonably priced with low side effects. As a source of natural antiviral chemicals, PSMs have the potential to be a viable alternative to standard pharmaceuticals, however, some PSMs can also be dangerous and expensive. Plant-based medications are becoming more and more popular on a daily basis. Crude plant extracts can be beneficial or dangerous depending on the amount employed; if processed correctly, higher activity may be demonstrated [78-80]. Crude extracts have also been shown to target numerous locations within a virion particle at the same time, according to the research. SARS-CoV-2 has not been tested for this. PSMs can disrupt intermediate metabolisms, interfere with DNA/RNA synthesis and function, obstruct normal cell communication (quorum sensing), and cause clotting of cytoplasmic constituents. SARS CoV antiviral activities are found in several plant metabolites [68]. The pathogenic process is influenced by plant-based compounds in a number of ways. When it comes to the human body's redox status, protein kinases, and transcription factors as well as adhesion molecules and cytokines, curcumin, for example, has anti-cancer and anti-proliferative capabilities. SARS CoV-2 anti-SARS CoV PSMs may be a valuable therapeutic target, according to an in-silico investigation. Modern methods for isolating lead compounds from crude extracts include maceration, percolation, decoction, reflux extraction, Soxhlet extraction, pressurized liquid extraction, supercritical fluid extraction, ultrasound extraction, microwave extraction, pulsed electric field extraction, enzyme extraction, hydro distillation, and steam distillation [81]. It is possible to identify new anti-SARS CoV-2 chemicals much more quickly using these techniques. Plant metabolomics is also being utilized to aid in the identification of new plant-based medicines [81-82].

Synthetic drugs and COVID-19 a hope in PSMs

Low molecular weight PSMs are produced by plants to protect themselves from a wide variety of herbivores and microorganisms. The use of these potent natural sources to treat a wide range of human ailments was common prior to the invention of allopathic medications. Plant metabolites, in particular, are being studied as potential lead molecules in the fight against human diseases because of microbial sickness resistance to allopathic treatments. Furthermore, about 35% of the world's medication supply is derived from natural plant or herb sources (worth 1.1 trillion US dollars). Researchers are attempting to produce new and current medicines derived from a variety of herbal therapies in order to overcome this microbial resistance battle. SARS CoV and SARS CoV-2 share significant characteristics (both of them belong to beta family, containing the same genetic material-RNA, and using the same receptor for viral attachment-ACE2, with an 86 percent identity and 96 percent similarity of genome, with almost the same pathogenesis). This means that previously identified antiviral plant metabolites against SARS CoV can be considered COVID-19 drug candidates. Antiviral medication research is currently constrained by the economic impact of viral infections around the world [83-85]. As opposed to other antiviral capabilities, several PSMs have already demonstrated anti-SARS CoV action. These findings point to the prospect of developing new medications and chemicals that are more customized to the needs of the patient. There is therefore a possibility of utilizing plants in the war against COVID19. Naturally occurring metabolites derived from a wide variety of plant species may be effective in the fight against COVID-19, according to findings from literature [86]. (Table 1)

**Table 1:** Name of the plants and the active metabolites used for COVID 19 treatment protocol

Name of the plant	Active metabolite	Action	Dosage
Asvagandha	Withaferin A	Anti covid 19 activity, Anti pyretic, Anti microbial, Antibacterial, Antioxidant, Immunomodulator,	Powder 3-6 gm
Guduchi	Tinosporin	Anti pyretic, Antioxidant, Immunomodulator	Fresh juice-10 -20 ml; Powder -2-6 gm
Kalamegha	Andrographolide	Antiviral, Antipyretic, Antiperiodic, Immune Enhancement, Hepatoprotective	Power 1-3 gm; fresh juice 5-10 ml
Tulasi	Bornylacetate, Cadinene	Anti viral, Antifungal, Antibacterial, adaptogenic (anti stress)	Fresh juice 10-20 ml
Tvak	Cinnamaldehyde	Anti complement activity, anti allergic activity	Powder 1 -3 gms
Adaraka	Alpha curcumene	Anti bacterial, Anti histaminic, Anti oxidant, Anti inflammatory	Fresh juice 5-10 ml; powder 1-2gm
Vasa	Vascine	Brochodilator activity, Haemostatic, advantages in attenuating the critical inflammatory stages of Covid 19	Leaf juice 10-20 ml
Sathi	Hedychenone	Anti bacterial, anti fungal, Anti inflammatory, Hypoglycaemic, 34 Vasodilator, relieves paraxysmol attack of dyspnoea, Tranquilizer	Powder : 1-3 gm
Puskaramula	Alantolactone,	Anti pyretic, Anti fungal, Anti microbial, Bacteriostatic, Fungistatic, Anti inflammatory, Anti histaminic, effective against bronchospasm, Hypoglycaemic, Anti anginal, hypolpidemic	Powder 1-3 gm

Action and structure of PSM

An antiviral component can be found in many different plant species. HIV, Dengue, Simian, and human rotavirus flavonoid molecules are widely disseminated low molecular weight phenolic HIV, Dengue, Simian, and bovine viral molecules. SARS CoV, Influenza virus, hepatitis B virus (HBV), herpes simplex virus (HSV), hepatitis C virus (HCV), vesicular stomatitis virus (VSV), and the Newcastle disease virus were all successfully treated with this compound, which demonstrated significant antiviral activity (NDV). Apigenin and quercetin, flavonoid-type medications, inhibited Mpro enzymes and were effective against SARS CoV virus particles, with IC50 values of 38,4 2.4 M and 23.8 M, respectively. SARS CoV-2 Mpro can be suppressed by flavonoid medications, according to an in-silico study. The heterocyclic ring structure of alkaloids is what distinguishes them from other naturally occurring chemical molecules. Some of them include tropanes (pyrrolidines), is quinoline purines, imidazole's, quinolizidine's and indoles (piperidines), as well as pyrrolizidines. There is a lot of promise in alkaloids for treating HIV-1, HSV-1, HSV-2, DNV, VSV, and influenza viruses (NDV). It was discovered that anti-SARS alkaloids such as emetine and 7-methoxy cryotolerance inhibit protease enzymes, RNA synthesis, and protein synthesis. SARS Cove's nucleic acid intercalating agent is tetrandrine, factionalize, catharanthine, and lycorine, which degrade nucleic acids and block spike and nucleocapsid proteins. SARS CoV and SARS CoV-2 antiviral activity was found in 10Hydroxyusambarensine and Crypto quindoline, two alkaloid compounds extracted from African medicinal plants. Chloroquine, an alkaloid derivative, has been found to have anti-SARS CoV-2 activity. Alkaloids in the form of PSMs could therefore serve as potential treatment targets for COVID-19. Saponins (amphipathic glycosides) are another class of PSMs found in plants that have antiviral activity against Newcastle disease virus (NDV), Simian (SA-11) virus, Murine norovirus (MNV), Feline calicivirus (FCV), RSV, VSV, HSV-1, HSV-2, HIV-1, Epstein-Barr virus (EBV), (SA-11), and human rotavirus (HCR3). Five terpenes derived from plant-produced carbon isoprene make up the most diverse and biggest class of PSM. Sesterterpenes, hemiterpenes, and sesquiterpenes are all types of terpenes [87-88]. The following viruses were shown to be resistant to them: In addition to HSV-1, poliovirus type 2, and vesicular stomatitis virus (VSV), bovine viral diarrhea virus, HSV-1, and HIV-1, HIV-2, SIV mac 251, SARS-CoV, and Influenza A and B viruses. There were ten diterpenes, two sesquiterpenes, and two triterpenes with IC50 values of 3-10 M. The terpene Ginkgolide A has been shown to have a strong inhibitory effect on the SARS CoV-2 protease enzyme in computer simulations [84]. Human Rotavirus, influenza A, HSV, Coxsackie B3, and VSV have been shown to be inhibited by carbohydrates (monosaccharides, disaccharides, polysaccharides, and oligosaccharides) [87]. Acyclovir is a Food and Drug Administration (FDA)-approved antiviral medication derived from Carissa edulis [88]. Herpes simplex virus, chickenpox, and shingles are among the most prevalent illnesses treated by this medication. It is possible to establish the group basic structure of a number of significant chemicals.

Obstacles in the way of future drug development insights

"Drug discovery from plant metabolites" refers to the extraction and purification of active components from conventional medications [89]. The chemical structures of natural plant products are complex and vary widely among species. Some PSMs are responsible for the biological activity of herbal medications whereas others belong to a different category altogether (see below). The molecular targets of PSMs are diverse. In the search for targets, researchers may turn to a variety of molecules, including enzymes, mediators, transcription factors, and even DNA. An in-depth knowledge of the chemical composition of plants allows for a more accurate appraisal of their potential and medical worth. There are many factors that contribute to the success of the transition of a bioactive molecule into a commercially viable pharmaceutical in recent decades [90,91]. This can be done with the help of cutting-edge technology and well-established techniques. The screening of natural items for the creation of new medications has been hastened by modern technology. High-throughput methods such as gas chromatography-mass spectrometry (GC-MS), nuclear magnetic resonance (NMR), infra-red (IR), high performance liquid chromatography (HPLC), and high-performance thin-layer chromatography (HPTLC) are essential for the development of strong and prudent lead compounds that can be progressed from a screening hit to a therapeutic candidate through structural elucidation and structure recognition confirmation. Innovative technology allows us to conduct research on novel compounds by leveraging computer programs and databases to identify common items as an important source for medication discovery. Innovative new technology is reshaping the study of herbal medicine and how it can be used [92,93].

However, a number of elements play a role in the translation of an intriguing chemical into a useful treatment alternative. These characteristics include the compound's exceptional pharmacokinetic profile, availability, bioavailability, and intellectual property. In vivo testing is critical before moving further with animal experiments or clinical trials in the future. Even if a medication shows promise in vivo, a poor pharmacokinetic profile may render it ineffective in animal model experiments. Target compounds are kept in close touch with cells when tested in humans, but in animal models, they may lose some of their bioactivity as a result of many processes [94,95]. The bioavailability of curcumin, for example, has prevented it from being commercialized as a medicine despite its numerous health benefits. Epigallocatechin gallate (EGCG), another fascinating medicinal candidate, has showed antioxidant, antihypertensive, anticancer, antibacterial, and anti-inflammatory potential but, like curcumin, has failed to obtain pharmacological classification for the same reason [96-99].

Researchers throughout the world are coming up with new approaches to deal with these issues. The bioavailability of a drug can be improved by administering it in a different way [99]. Andrographolide, an anti-inflammatory chemical, has a twofold increase in bioavailability when given intravenously rather than orally [100]. Adjuvant systems, drug delivery systems, nano-formulation of a medicine, and structural analogy change are further methods for increasing a target chemical's bioavailability [100,101]. Pharmacokinetic characteristics, such as absorption, distribution, metabolism, and excretion, can potentially be altered to improve a substance's potential to be used as a therapeutic agent. It is critical that the World Health Organization (WHO), FDA, European Medicines Agency (EMA), World Trade Organisation (WTO), International Conference on Harmonization (ICH), World Intellectual Property Organization (WIPO) collaborate on the development of specific protocols for the discovery of novel bioactive compounds [101,102]. Indeed, there is an urgent need for specific protocols for the discovery of novel bioactive compounds, and related organizations, companies, and agencies must collaborate on this effort. A wide range of serious illnesses brought on by lethal viruses have already showed promise in the treatment of plant-derived medicines, which should be tested against SARS-CoV-2.

SARS CoV-2 has been shown to have seven major pharmacological targets. Molecular docking can save both time and money when screening PSMs for drug establishment. MERS has been shown to be effectively vaccinated against using animal models, target antigens, and vaccine candidates generated using computational biology [103,104]. There is, however, a lack of in-depth research on PSMs as an alternative to pharmaceuticals. SARS-CoV-2 draggability investigations have become a time-saving method by recommending possible antiviral plant metabolites or screening. Without a clinical trial pathway in place, many promising candidates would not be able to move forward. PSMs will never be developed into modern medicines and will remain in journals or herbal concoctions. In the fight against antibiotic resistance, the plant is unquestionably an underappreciated source of new bioactive chemicals. As the amount of plant metabolites increases, PSM decryption progresses slower. SARS-CoV-2 secondary metabolites can be produced in a timely and ecologically safe manner using a biotechnological technique [105]. Secondary metabolic pathways require an in-depth knowledge of the genes and proteins involved. In food and drug research, omics approaches (transcriptomics, proteomics, and metabolomics) are essential. Genetic engineering of plant metabolites can assist in the manufacturing of a particular medication [106, 107]. The quality of natural products must also be monitored. Consequently, laboratory assistance, qualified employees, and financing are all necessary for the discovery of natural resource-based drugs.

## Conclusions

The review puts a light on how researchers from all around the world are analyzing the B1.1.529 Omicron strain of SARS-CoV-2, about its discovery in Botswana and reporting in India. Many plants secondary metabolites are mentioned in our review and may be considered for high-quality IRB-approved clinical research RCTs. These compounds interfered with the essential machinery involved in the pathogenesis and reproduction cycle of the coronavirus. In vitro, in vivo, and in silico, numerous anti-SARS CoV and anti-SARS CoV-2 plant-derived compounds have been discovered. Antiviral bioactive substances can be found in abundance in plants. Plants with potential antiviral efficacy against SARS Cove and MERS may be an attractive treatment option for SARS CoV-2. We have reported here on nine plants that have antiviral activity against a variety of DNA/RNA viruses. The possible effects of such alternatives should be subjected to rigorously designed clinical research trials before being incorporated into practice
